# Antiretroviral activity of Amazonian plants

**DOI:** 10.1186/1742-4690-8-S2-P87

**Published:** 2011-10-03

**Authors:** Elida CG da Mata, Magda CA Gonçalves, Jorge FO Segovia, Roberto M Bezerra, José CT Carvalho, Luís IB Kanzaki

**Affiliations:** 1 Laboratory of Bioprospection, University of Brasília, DF, Brasília, Brazil, CEP 70.910-900; 2 Secretary of Science and Technology, Macapá, AP, Brazil, CEP 68.901-335; 3 Brazilian Agricultural Research Corporation, Macapá, AP, Brazil, CEP 68903-419; 4 Laboratory of Drugs, Federal University of Amapá, Macapá, AP, Brazil, CEP 68902-280

## Background

The Amazon region displays a rich and diverse biota encompassing more than 50,000 botanical species [1]. A few medicinal plants commonly utilized by local people has been studied concerning its pharmacological properties. New antiretroviral drugs are on demand, mainly in developing countries and particularly in Brazil, which exhibit an exuberant biota, it is mandatory to rationally explore its immense and diverse floristic potential for medicinal purposes [2].

## Materials and methods

Plants representatives of the Apocynaceae, Rubiaceae, Fabaceae, Caesalpiniaceae, Ochnaceae, Clusiaceae, Arecaceae, Chrysobalanaceae and Olacaceae families were collected in distinct geographic locations in the Amapa state, in the northern region of Brazil. Aqueous extracts of bark, leaves and fruits were prepared from the above botanical species. An established lymphoblastic cell line, H9, cultured in RPMI medium supplemented with fetal bovine serum and antibiotics was utilized to replicate the Simian Immunodeficiency Virus, strains SIVagm155-4 and SIVmac186. Previously to SIV infection, H9 cells were cultured in a 48 plate at 5.95X105 density per well in 500 uL and treated in high, median and low concentration of each aqueous plant extract and citotoxicity was evaluated by the WST assay. After determining the inocuous aqueous extract concentration, cells were treated with the plant extract and after 48 hours, cells were washed and infected with 599.64 pg/mL (SIVp27) of SIV. After 96 and 144 hours post infection, samples of cell supernatant were collected of each well and assayed for viral replication by measuring SIVp27 utilizing the SIVp27 Antigen Capture Assay (Advanced Bioscience Laboratory, Inc.) [3].

## Results

Of all plants aqueous extracts tested, 8 reduced SIV replication but just two, representatives of Fabaceae and Chrysobalanaceae families, did reduce virus replication but did not reduce cell density. Also, besides the antiretroviral activity found, proliferative and citotoxic activity was detected among the plants herein studied.

## Conclusions

Ongoing studies aim at the identification of plant extract fractions exhibiting antiretroviral, proliferative and cytotoxic activities in order to plan future research work to develop new drugs.
